# Challenges in Forecasting Antimicrobial Resistance

**DOI:** 10.3201/eid2907.230489

**Published:** 2023-07

**Authors:** Mamoon A. Aldeyab, William J. Lattyak

**Affiliations:** University of Huddersfield, Huddersfield, UK (M.A. Aldeyab);; Scientific Computing Associates Corp., River Forest, Illinois, USA (W.J. Lattyak)

**Keywords:** predictive modeling, infections, antimicrobial resistance, bacteria, United Kingdom, United States

**To the Editor:** We read with interest the article by Pei et al. (*1*), which discussed challenges in forecasting antimicrobial resistance (AMR) and emphasized the need for improved AMR predictive intelligence. We complement the authors on their findings and share our experience with a threshold-logistic modeling concept that we recently introduced to improve understanding of the relationship between antimicrobial drug use thresholds and incidence of resistant pathogens and a threshold transfer function model that can be used to project AMR prevalence (*2*,*3*).

AMR modeling and forecasting are challenging because of the evolutionary ability of pathogens to adapt to changing environmental conditions. To be useful, a model should address time-varying effects of explanatory variables on response function and changes in the structural relationship between predictor and target variables. Concepts such as threshold transfer function modeling that includes autoregressive moving average components can partially address those issues. Model recalibration is essential and dictated by breakdown in predictive ability. Model parameters can be reestimated after recording new observations. Model structure does not change, but parameter estimates are updated to reflect new information. If statistical significance of a parameter falls below a defined confidence level, model structure might need modification. In practice, model estimation and assessment can be automated. Forecasting model accuracy can also be automated by comparing ongoing performance to baseline accuracy. When model or predictive performance degradation is flagged, a more comprehensive model recalibration is dictated. The time between model calibrations is unknown and depends on the stability of identified relationships in the model and degree to which evolutionary changes in AMR are observed. Further real-world testing is required to determine other factors that can explain resistance and define thresholds, find optimal interventions to reduce antimicrobial drug use to identified thresholds, and assess feasibility of implementing those interventions in daily clinical practice.
